# Estimates of child deaths prevented from malaria prevention scale-up in Africa 2001-2010

**DOI:** 10.1186/1475-2875-11-93

**Published:** 2012-03-28

**Authors:** Thomas P Eisele, David A Larsen, Neff Walker, Richard E Cibulskis, Joshua O Yukich, Charlotte M Zikusooka, Richard W Steketee

**Affiliations:** 1 Department of Global Health Systems and Development, Tulane University School of Public Health and Tropical Medicine, 1440 Canal Street, Suite 2200, New Orleans, LA 70112, USA; 2 Department of International Health, Johns Hopkins Bloomberg School of Public Health, 615N. Wolfe Street, Baltimore, Maryland 21205, USA; 3 World Health Organization, Global Malaria Programme, Geneva, Switzerland; 4 HealthNet Consult, Kampala, Uganda; 5 Malaria Control and Evaluation Partnership in Africa (MACEPA), a program at PATH, 2001 Westlake Ave, Seattle, WA, USA

## Abstract

**Background:**

Funding from external agencies for malaria control in Africa has increased dramatically over the past decade resulting in substantial increases in population coverage by effective malaria prevention interventions. This unprecedented effort to scale-up malaria interventions is likely improving child survival and will likely contribute to meeting Millennium Development Goal (MDG) 4 to reduce the < 5 mortality rate by two thirds between 1990 and 2015.

**Methods:**

The Lives Saved Tool (LiST) model was used to quantify the likely impact that malaria prevention intervention scale-up has had on malaria mortality over the past decade (2001-2010) across 43 malaria endemic countries in sub-Saharan African. The likely impact of ITNs and malaria prevention interventions in pregnancy (intermittent preventive treatment [IPTp] and ITNs used during pregnancy) over this period was assessed.

**Results:**

The LiST model conservatively estimates that malaria prevention intervention scale-up over the past decade has prevented 842,800 (uncertainty: 562,800-1,364,645) child deaths due to malaria across 43 malaria-endemic countries in Africa, compared to a baseline of the year 2000. Over the entire decade, this represents an 8.2% decrease in the number of malaria-caused child deaths that would have occurred over this period had malaria prevention coverage remained unchanged since 2000. The biggest impact occurred in 2010 with a 24.4% decrease in malaria-caused child deaths compared to what would have happened had malaria prevention interventions not been scaled-up beyond 2000 coverage levels. ITNs accounted for 99% of the lives saved.

**Conclusions:**

The results suggest that funding for malaria prevention in Africa over the past decade has had a substantial impact on decreasing child deaths due to malaria. Rapidly achieving and then maintaining universal coverage of these interventions should be an urgent priority for malaria control programmes in the future. Successful scale-up in many African countries will likely contribute substantially to meeting MDG 4, as well as succeed in meeting MDG 6 (Target 1) to halt and reverse malaria incidence by 2015.

## Background

Malaria is a major contributor to child mortality in sub-Saharan Africa [1,2]. Fortunately, vector control through insecticide-treated mosquito nets (ITNs) and malaria prevention during pregnancy through ITNs and intermittent prevention therapy (IPTp), have been shown to significantly reduce the burden of malaria from carefully conducted trials [3-6]. A recent analysis of 29 national-level cross-sectional datasets in Africa that assessed the association between ITN household possession and all-cause post-neonatal child mortality showed the effect of ITNs under routine programme conditions to be nearly identical to, if not greater than, the efficacy observed in trials [7].

Since the launch of the Roll Back Malaria Partnership (RBM) in 1998, many countries have worked to expand coverage of these proven malaria prevention interventions. Funding from external agencies for malaria control in Africa has increased by a factor of 40 since 2000, reaching more than US$1.47 billion in 2009 [8,9]. As a result of both increased funding from external agencies and increased attention to malaria by national governments, national coverage levels of malaria prevention interventions, namely ITNs and IPTp, have increased dramatically across sub-Saharan Africa. This unprecedented effort to scale-up malaria interventions is likely improving child survival and will likely contribute substantially to meeting Millennium Development Goal (MDG) 4 (Target 1) to reduce the < 5 mortality rate by two thirds between 1990 and 2015.

Unfortunately, vital registration data are generally not available in most malaria-endemic countries for ascertaining changes in malaria-specific and all-cause child mortality [10-13]. Other methods for measuring child mortality in Africa, such as demographic surveillance systems and household surveys, have serious limitations for producing timely trends in malaria mortality at the country level. Thus, real-time tracking of changes in child mortality, especially malaria-specific mortality, presents serious challenges.

Mathematical modelling has been recommended as a method to gain perspective into the possible impact of malaria interventions [14]. Previous modelling estimates have suggested that approximately 691,000 all-cause child deaths could have been prevented in the year 2000 alone had universal coverage of ITNs been achieved, and 22,000 child deaths could have been prevented in 2000 had universal coverage of IPTp been achieved [15]. Another cost-effectiveness modelling approach estimated that approximately seven million additional disability adjusted life-years (DALYs) could be averted by adding universal ITN coverage on top of universal access to malaria treatment with artemisinin-based combination therapy (ACT) [16].

To estimate the impact that improved access to effective child survival interventions have on reducing child mortality, estimates of the relative reduction in child mortality of empirically proven child survival interventions have been linked to the population coverage of such interventions. This effort culminated in estimates of the impact of scaling-up interventions in *The Lancet *Series on Child Survival [15], Neonatal Survival [17], and Maternal and Child Under-nutrition [18,19]. A central component to that work was the development of a model to estimate the reduction in child mortality that could be achieved with expanded coverage of effective child survival interventions. This model, now referred to as the Lives Saved Tool (LiST), has continued to be refined to allow retrospective estimation of deaths prevented by intervention scale-up. The use of LiST to retrospective model estimates of neonatal and child deaths prevented from the scale-up of packages of child survival interventions have been shown to yield reasonably reliable estimates when compared to measured changes in mortality across various settings, including neonatal mortality in South Asia [20], all-cause < 5 year child mortality from a broad package of child health interventions in West Africa [21], and all-cause < 5 year child mortality in Bangladesh [22].

It is known that the coverage of malaria prevention interventions, especially vector control through ITNs, has increased dramatically over the past 10 years in Africa, with the bulk occurring since 2005. However, it remains unknown what impact this increased access to proven malaria prevention intervention has had on child mortality over the past decade. The LiST model was used to approximate the likely impact that malaria prevention intervention scale-up has had on child malaria mortality over the past decade (2001-2010) across 43 malaria-endemic countries in Africa. Estimates of the cost effectiveness of ITNs are estimated from 2006-2009 during the peak scale-up. The model was also used to estimate the potential number of malaria deaths that could be prevented from additional scale-up of malaria prevention interventions to the RBM universal coverage target of 100% from 2012 through 2015 [23]. Unfortunately, due to difficulties with the definition and matching coverage estimates of prompt treatment of childhood fevers with ACT to estimates of the efficacy of ACT at preventing child deaths [24], the current analysis does not include an estimate of the child deaths prevented from malaria treatment.

## Methods

**Figure 1:**
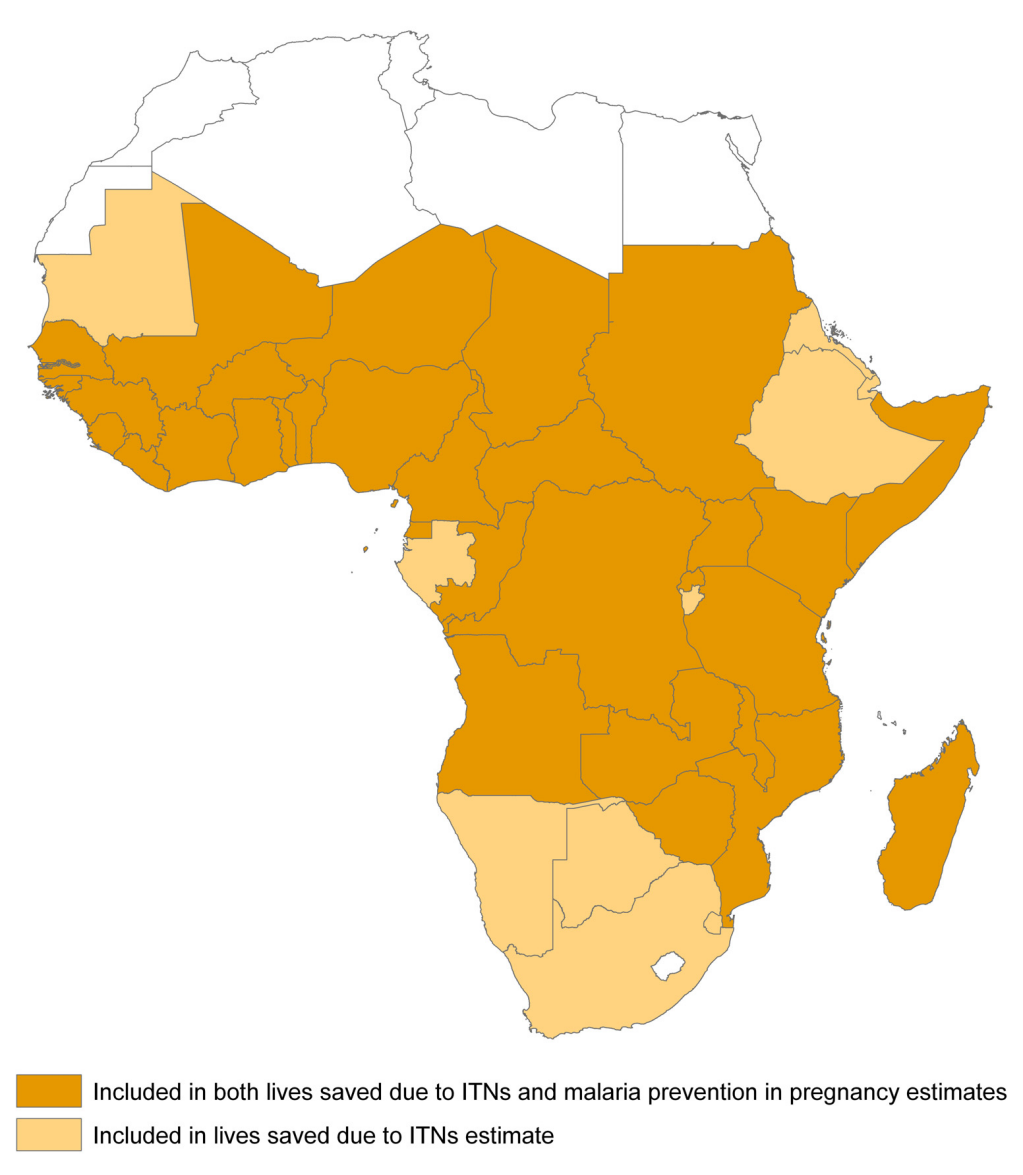
**Malaria-endemic countries included in estimates of malaria intervention scale-up and resultant malaria-caused child deaths prevented 2001-2010**.

This analysis, using the LiST model, focused on estimating the impact of ITNs for preventing post-neonatal (one-59 months of age) child malaria deaths from 2001-2010, as compared to a baseline of 2000. A baseline of 2000 was chosen as this is the year prior to any scale-up of ITNs or malaria prevention in pregnancy interventions in most African countries. Forty-three countries in sub-Saharan Africa are included in this analysis; 43 for the ITN analysis and 32 for the malaria prevention in pregnancy analysis (Figure 1) [25].

This analysis of malaria prevention during pregnancy (with either IPTp or ITNs) for preventing all-cause < 5 child deaths through prevention of low birth-weight (LBW) was limited to 32 countries in Africa with stable malaria transmission. These countries accounted for 88% of the population in SSA at risk of malaria and 90% of the malaria-caused child deaths in 2000 in SSA [25,26]. A list of excluded countries from the analyses, as well as the rationale for exclusion, can be found in Additional file 1.

The LiST model is based on the earlier work on effectiveness of interventions developed by WHO and UNICEF's Child Health Epidemiology Reference Group (CHERG) and RBM's Monitoring and Evaluation Reference Group (MERG). The model starts with estimates and assumptions within each country on the profiles for population and population growth, under-five cause of death structure, disease-specific mortality rates, and current coverage estimates of key child survival interventions [27]. The model used in this analysis and accompanying documentation can be downloaded [28].

The model estimates child deaths prevented (within specific cause of death categories) due to intervention scale-up within a specified country as a function of three primary parameters, as outlined in details below: 1) the number of child deaths by cause projected to occur in each year (including population growth parameters over time); 2) the protective effect (PE) on cause-specific mortality (PE = 1-relative risk*100) for each intervention being scaled-up; and 3) increases in population coverage of each intervention. The model computes the number of deaths prevented by cause each year, accounting for population growth, as the difference between the estimated deaths that occur with intervention scale-up and the estimated deaths that would have occurred without intervention scale-up beyond the coverage at a baseline year. Further details of the LiST model estimation methods are presented in the Additional file 1.

### Parameter 1- within country estimates of cause-specific child deaths

Within the LiST model, the total number of < 5 child deaths for the baseline year of 2000, by age, is based on estimates of < 5 mortality produced by the UN Inter-agency Group for Child Mortality Estimation (IGME) [29]. The number of post-neonatal child malaria deaths in our baseline year of 2000 was estimated as the proportion of child deaths one-59 months attributable to malaria [26], multiplied by the total number of all-cause < 5 child deaths one-59 months in the year 2000. Based on this method, the LiST model started with 870,928 malaria-caused deaths one-59 months in 43 African countries and 1,034,828 all-cause neonatal deaths in 32 African countries in the baseline year of 2000 (Additional file 2).

### Parameter 2- estimates of intervention effectiveness

As described in detail elsewhere, the PE of ITNs for preventing post-neonatal child malaria deaths has been estimated to be 55% (range 49-60%) based on a systematic review and meta-analysis of three trials [30].

Malaria in pregnancy increases the risk of LBW, resulting primarily from intrauterine growth retardation (IUGR) in areas of stable *P. falciparum *malaria transmission [31], while LBW has been demonstrated to be a significant risk factors for neonatal and infant mortality [32-34]. The PE of malaria prevention during pregnancy for preventing LBW has been estimated to be 35% (95% confidence interval [CI] 23-45%) during the first two pregnancies in malaria endemic areas based on a systematic review of 2 ITN trials and 3 IPTp trials conducted in 1998 and 2002 in Kenya and 2004 in Mozambique, after the onset of sulphadoxine-pyrimethamine (SP) resistance [30]. The effect of malaria prevention interventions during pregnancy on LBW in the LiST model acts solely through intrauterine growth retardation (IUGR). IUGR in the LiST model acts mostly through neonatal mortality, increasing the risk of dying during this period due to diarrhoea [RR = 2.0], sepsis/pneumonia (RR = 2.0), and asphyxia (RR = 2.3). During the post-neonatal period, IUGR slightly increases the risk of dying due to measles, malaria, diarrhoea and pneumonia [18,35], via links between IUGR and stunting. In this analysis, the effect of IPTp and ITNs acted only on child deaths from the first two pregnancies of women in each country.

### Parameter 3- changes in coverage of malaria prevention interventions

Estimates of household ITN coverage used in this analysis were based upon previous publications that modeled the proportion of household with ≥ 1 ITN each year in each country in Africa based on survey data and ITN procurement and distribution data, with uncertainty [35,36]. These estimates are adjusted for each country's population at-risk for malaria such that coverage reflects the assumption that all nets are received and owned by households in malaria endemic areas of the country.

The LiST model uses ITN household possession instead of ITN use by children because the trials from which 55% PE was derived all used intention-to-treat analyses, meaning the estimated effects (relative risk) were based on whether or not a child lived in a village that had high ITNs household possession. Results from the analysis of the association between ITNs and all-cause post-neonatal mortality across 29 cross-sectional datasets in Africa showed very little difference in the effect regardless of whether ITN household possession or ITN use by children the previous night was used [7], further supporting the use of household ITN possession instead of child use.

The impact of malaria prevention during pregnancy on LBW and subsequent < 5 child survival was estimated using the higher of the following two coverage indicators: 1) proportion of pregnant women using an ITN the previous night or; 2) the proportion of women who had a live birth in the past two years who received ≥ 2 doses of SP during an ante-natal care (ANC) visit. The authors are unaware of published yearly national estimates of malaria protection during pregnancy. Estimates of coverage of malaria prevention during pregnancy used in this analysis were derived from nationally representative household surveys, including the Demographic and Health Survey (DHS), the Multiple Indicator Cluster Survey (MICS), the Malaria Indicator Survey (MIS) and the AIDS Indicator Survey (AIS), as summarized below and described in detail elsewhere (Additional file 1). Because the majority of malaria deaths and the burden of malaria in pregnancy is concentrated in rural areas [1,37], and as intervention coverage in rural areas has often lagged behind urban areas in many countries in Africa [38], the level of malaria prevention during pregnancy in rural areas was used for estimating the malaria deaths prevented from malaria prevention during pregnancy scale-up at the national level.

If there was no survey data point for IPTp/ITN coverage in 2000, coverage was assumed to be 0%. Linear interpolation was used from the first year of measured IPTp/ITN coverage, or from 0% coverage in 2000, to the next available survey point estimate. This slope was then used to inform the increase for years beyond the most recent household survey through 2010. In the case that there was only one household survey in the country, coverage was assumed to be 0% in the year IPTp was implemented as policy, and a linear slope was calculated from that year to the household survey data point. For countries projected to reach ≥ 80% coverage, maximum coverage estimates were limited to the proportion of women attending ANC at least once in last nationally representative household survey (see Additional file 1 for details on methods, as well as Additional files 3 and 4 for results).

### Key assumptions of this LiST analysis

The LiST model analysis conducted here has several important assumptions. First, the LiST model assumes that the PE of interventions is constant across each country in this analysis. While the PE of ITNs interventions varies across level of intervention coverage in the population, child age and malaria transmission level, the PE of ITNs used in this analysis is assumed to represent a valid estimate of the mean effect across these effect modifiers. Results from an analysis of 29 cross-sectional country datasets in Africa since 2000 with varying levels of ITN coverage and across varying levels of malaria transmission showed the average effectiveness of ITN under programme conditions to be very similar to the trials for reducing all-cause one-59 month child mortality, which helps support the use of a fixed 55% PE for ITNs used here [39]. A previous validation study of the LiST model against measured reductions in all-cause child mortality following the scale-up of ITNs, across a range of transmission settings, further validates the use of the fixed PE of 55% for ITNs used here [40]. Second, while there is evidence that malaria acts as a significant risk factor for dying from conditions other than malaria during childhood [41], the LiST model constrains deaths prevented by malaria control within the envelope of malaria-attributable < 5 deaths. Deaths associated indirectly with malaria as a contributory cause were not counted, although the ITN trials had shown that this indirect contribution of malaria may be an equally important additional contribution of malaria's overall effect on < 5 mortality as the directly malaria-attributed deaths.. Third, intervention coverage for a given year is assumed to be a valid estimate for the mean coverage over the 12-month period. Fourth, due to a lack of data to quantify effect modification, the model does not account for any possible synergistic or saturation effect between ITNs and IPTp for preventing child deaths [42]. And lastly, coverage by a malaria prevention intervention is assumed independent from coverage by another malaria prevention intervention, which is likely not true.

### Calculation of percent reduction in malaria deaths

Percent reduction in malaria deaths were estimated by dividing the number of malaria deaths estimated to occur with intervention coverage scale-up in a given year (or over a period of years) by the number of malaria deaths estimated to have occurred in that year (or over that period) had no intervention scale-up occurred from 2000.

### Uncertainty about LiST estimates

Uncertainty bounds about total estimated post-neonatal child malaria deaths prevented from ITNs 2001-2010 were based on a non-probabilistic sensitivity analysis of the uncertainty of the three primary model parameters. This resulted in a worst-case (i.e. low-impact) and best-case (i.e. high-impact) scenario, with the worst-case using the lower bound of estimated post-neonatal malaria deaths in 2000 in each country [26], the lower bound of the PE for ITNs (49%) [30], and the smallest net increase (slope) in ITNs scale-up 2001-2010 for each country based on the uncertainty of the coverage estimates by Flaxman and colleagues [35]. The best-case scenario resulted from using the upper bound of estimated post-neonatal malaria deaths in 2000 in each country, the upper bound of the PE for ITNs (60%), and the largest net increase (slope) in ITNs scale-up 2001-2010 for each country based on uncertainty about the coverage estimates (Additional file 1 for details on methods).

Uncertainty bounds about total estimated < 5 deaths prevented from malaria prevention during pregnancy 2001-2010 were obtained based on a similar non-probabilistic sensitivity analysis of the uncertainty of two primary model parameters: lower and upper bound of the estimated PE of malaria prevention in pregnancy on preventing LBW [30], and the smallest and largest net increases in intervention coverage changes within each country from 2001-2010 based on sampling errors (see Additional file 1 for details on methods, as well as Additional file 4 for resultant uncertainty of malaria prevention in pregnancy scale-up for each country 2000-2010). This resulted in a worst-case and best-case scenario as described above.

### Cost effectiveness

A review of existing literature on the costs and cost effectiveness of ITN delivery [ITN or long-lasting ITN (LLIN)] in Africa since 2005 was conducted in PubMed and the grey literature; 13 studies were identified and included [43-55]. Costs of ITN programmes were separated into delivery and commodity components, and used to estimate a median cost of ITN delivery, and of ITNs procured for programmes. Total costs were then calculated over the period 2006-2009 following previous methodological guidelines [56,57]. Costs were combined with estimates of lives saved and Disability Adjusted Life Years (DALYs) over this time period to produce cost effectiveness results. A one-way sensitivity analysis was also conducted (see Additional file 1 and 5 for additional details).

## Results

### The 2001-2010 decade

**Figure 2:**
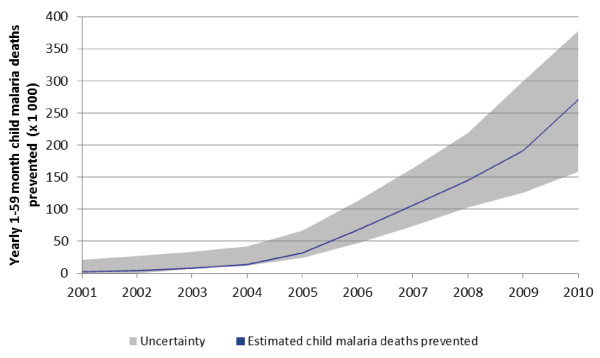
**Number of yearly malaria-caused child deaths prevented by malaria prevention interventions scale-up 2001-2010**.

Using the LiST model, this analysis estimated that malaria prevention intervention scale-up over the past decade has prevented 842,800 (uncertainty: 562,800-1,364,600) malaria-caused child deaths across 43 malaria-endemic countries in Africa, compared to a baseline of the year 2000 (Figure 2). Over the entire decade, this represents an 8.2% decrease in the number of malaria-caused child deaths that would have occurred over this period had malaria prevention coverage remained unchanged since 2000 coverage levels. The biggest impact occurred in 2010 with a 24.4% decrease in malaria-caused child deaths compared to what would have happened had malaria prevention interventions not been scaled-up beyond 2000 coverage levels.

Based on the estimated coverage of household possession of ≥ 1 ITN [36,37], it is estimated that scale-up of ITNs prevented 831,100 (uncertainty: 555,800-1,347,200) post-neonatal child malaria deaths across the 43 countries included in this analysis from 2001 through 2010 (Additional file 2). In Nigeria alone, which had an ITN coverage increase from 0 to 45% over this period, 165,700 (uncertainty: 96,800-240,000) post-neonatal child malaria deaths were estimated to have been prevented. Other main contributors to the total post-neonatal child malaria deaths prevented over the past decade, due primarily to substantial ITN scale-up and their large population size, include the Democratic Republic of the Congo, Ethiopia, Mali, Tanzania and Uganda, which total 286,300 (uncertainty: 205,000-429,800) child malaria deaths prevented compared to 2000.

**Figure 3:**
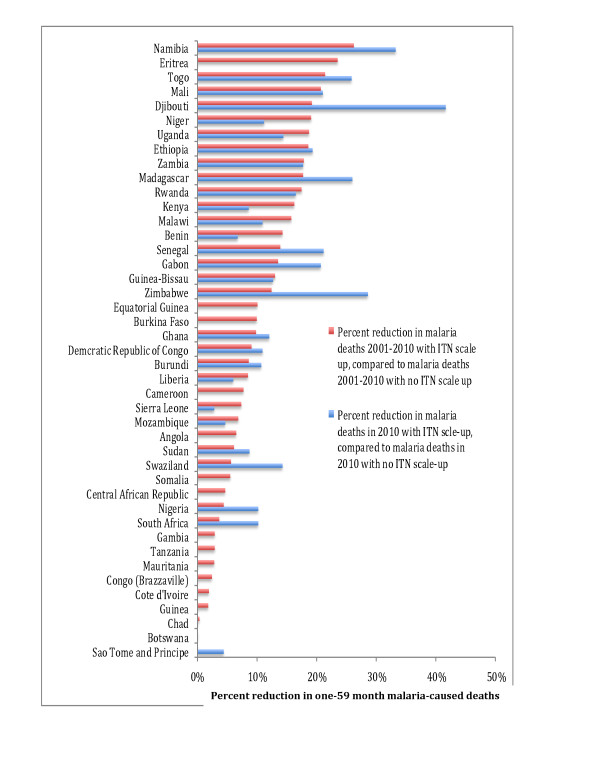
**Percent reduction in malaria-cased child deaths one-59 months**.

From ITN coverage scale-up 2001-2010, Namibia was estimated to have the largest percentage decline in post-neonatal malaria deaths from 2001-2010 with a 26% decline, while accounting for population growth, followed by Eritrea (24% decline), Togo (21% decline), Mali (21%) and Djibouti (19%) (Figure 3). The five African countries analysed with the largest number of malaria deaths in 2000 had the following percentage declines in post-neonatal malaria deaths from 2001-2010: Nigeria (4% decline), Democratic Republic of Congo (DRC) (9% decline), Uganda (14% decline), Tanzania (3% decline) and Southern Sudan (6% decline). For many countries, the bulk of the decline in malaria-caused mortality occurred in 2010 following rapid ITN scale-up (Figure 3 and Additional file 2 Table 1). Djibouti was estimated to have the largest percentage decline in post-neonatal malaria deaths in 2010 with a 42% decline, followed by Namibia (33% decline), Zimbabwe (29% decline), Togo (26% decline), Madagascar (26% decline), Mali (21%), Senegal (21% decline) and Gabon (21% decline).

**Figure 4:**
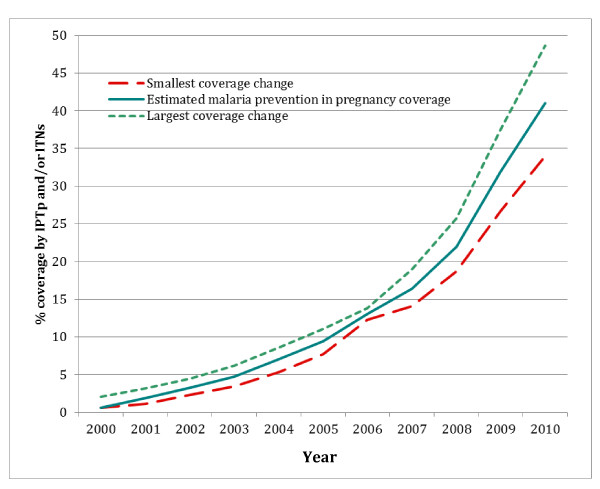
**Proportion of pregnant women in rural areas protected by malaria prevention interventions (IPTp and/or ITNs) across 32 countries in sub-Saharan Africa 2000-2010**.

Across the 32 countries with stable malaria transmission included in this analysis, it is estimated the proportion of pregnant women protected by either IPTp or ITNs in rural areas increased from a mean of 0.7% in the year 2000 to 41.8% in 2010 (Figure 4). It is estimated that from 2001-2010 IPTp and ITNs used during pregnancy prevented a total of 11,700 (uncertainty: 7,000-17,500) child deaths from LBW as a result of malaria in pregnancy, as compared to a baseline year of 2000 (Additional file 2).

### Cost effectiveness 2006-2009

This analysis estimated the median cost of delivery of an ITN to be US$1.64 (Table 1 in Additional file 5). There was some difference between subgroups of delivery systems (median US$1.34 for mass campaigns and US$3.96 for retail distribution). This analysis estimated the median procurement cost of an ITN to be US$5.44. When discounted over a three-year period, one year of ITN ownership was valued at US$1.85. The number of ITNs available in each year in all of the relevant areas of sub-Saharan Africa was estimated to be approximately 130 million, yielding approximately 520 million available net years over the period 2006-2009. Large differences in availability of nets arose from different assumptions of net lifetimes although this did not result in large differences in cost-effectiveness estimates (See Table 2 in Additional file 5). The total discounted cost of all ITNs and their delivery was estimated to be approximately US$1.3 billion. Based on predictions from the LiST model, the number of discounted lives saved using vector control methods over the period (2006-2009) was 475,800, resulting in an estimated US$2,770 per life saved. Lives saved over this period translate into approximate 11.9 million DALYs, resulting in an estimated US$111 per DALY averted. Results were particularly sensitive to variation in the price of an ITN and the number of nets delivered, but the intervention remained very attractive in low-income country settings under all scenarios tested (for the full results of the sensitivity analysis see Additional file 5).

### Estimates for 2011-2015

**Figure 5:**
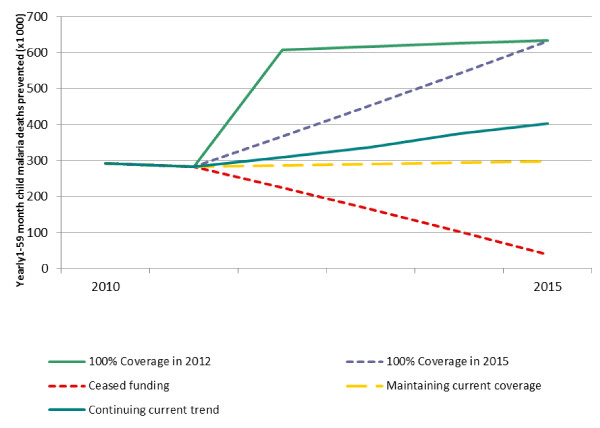
**Number of yearly post-neonatal child malaria deaths prevented by ITNs, according to different scale-up scenarios 2011-2015**. Continuing current trend was calculated using the slope between the most recent survey estimate and the earliest survey estimate (solid blue line). Achieving 100% coverage by 2015 assumes linear ITN coverage increases to 100% from estimated coverage in 2010 (dotted purple line). Maintaining coverage assumes estimated coverage in 2010 continues through 2015 (dashed orange line). Ceased funding was calculated assuming that ITNs last three years and have a net discard rate of 4% each year [36] (dashed red line).

Five possible ITNs scale-up scenarios beyond 2010 were examined (Figure 5). In the case of rapid scale up from estimated 2011 coverage levels to universal coverage (100%) by the end of 2012 and maintained through 2013-2015, it is estimated that 2.77 million post-neonatal child deaths could be prevented for this five-year interval. Achieving such universal coverage of ITNs would result in a 54% reduction in malaria-caused child mortality compared to maintaining 2000 coverage levels, after accounting for population growth; this translates to a decline in the 2015 all-cause < 5 mortality rate by 11.2% due solely to ITNs. With linear scale-up to universal coverage by the end of 2015 from 2011 levels, it is estimated 2.28 million child deaths could be prevented for this five-year interval, as compared to maintaining 2000 coverage levels. If current country scale-up trends 2000-2011 are continued on the same slope through 2012-2015, it is estimated that an additional 1.71 million child deaths could be prevented from 2011-2015, as compared to 2000 coverage levels. Alternatively, if current country coverage is stabilized at 2011 levels, it is estimated that 1.45 million child deaths could be prevented. However, if funding ceased and vector control strategies were no longer available and long-lasting ITN (LLIN) coverage decreased over the five-year interval, assuming LLINs last about three years [59], as many as 640,400 children would die as a result during this period, compared to maintaining 2011 coverage until 2015.

## Discussion

This analysis used the LiST model to estimate the effect of malaria prevention scale-up over the past decade on reductions in malaria-caused child mortality across 43 malaria-endemic countries in Africa. This analysis estimates that from 2001 through 2010, scale-up of malaria interventions likely prevented nearly one million (842,800) child deaths across these countries, corresponding with a mean decrease in malaria-caused deaths by 8.2% over the entire period. This represents saving 230 children from dying from malaria each day over the past decade as a result of malaria prevention scale-up, increasing from seven children saved each day in 2001 up to 741 saved each day in 2010.

Compared to a baseline of 2000 in the 43 countries analysed here, it is estimated that malaria-caused deaths in children one-59 months decreased by 24.4%, after accounting for population growth. While our estimates suggest that no country achieved the RBM goal of a 50% reduction in malaria deaths in 2010 compared to 2000, after accounting for population growth, it is estimated that 8 countries achieved ≥ 20% reduction from 2001-2010: Djibouti, Namibia, Zimbabwe, Madagascar, Togo, Senegal, Mali, and Gabon.

In this analysis, the majority of the child deaths prevented occurred since 2006 once scale-up of ITNs accelerated across the continent. ITNs accounted for 99% of the estimated child deaths prevented in this analysis, which did not include case management with ACT or IRS. Nearly 2.27 million child deaths could be prevented from 2011-2015 if universal coverage (100% of households) with ITNs is achieved by 2015, resulting in halving (54%) child malaria deaths compared to 2000 coverage levels after accounting for population growth. However, if funding ceased and the population coverage level of vector control strategies waned, nearly more than a half million children would die as a result. Rapidly achieving and then maintaining universal coverage of ITNs should be an urgent priority for malaria control programmes. Millions of child deaths can be prevented.

Though the estimated cost per DALY averted (US$111) and life saved (US$2,770) using ITNs are slightly higher than in some recent studies [46,48,58], they still fall into a range feasible for low-income countries. These estimates of cost are based on deliveries of nets on a continental scale, and thus incorporate programme leakage, wastage, delayed delivery to households, and targeting of populations other than children and pregnant women, making them conservative as compared to those derived directly from trials. Despite this conservative approach, the intervention meets the criteria for being very cost-effective in a less developed country established by the WHO Commission on Macroeconomics and Health; one DALY averted for less than average per capita income (for the countries included in this analysis the median GNI per capita was USD 590 in 2009 (Inter Quartile Range (USD 410-1,215) [59]. The scale-up of ITNs for malaria in sub-Saharan Africa has clearly been a sound investment in health.

There are number of important limitations to this analysis that must be considered when interpreting these results. First, other than the input parameter for child mortality for each country in 2000 that is based on IGME estimates, which are based on an analysis of all available mortality data for the given country [29], child mortality was never directly measured using this modelling approach. Second, this analysis is based on a number of key assumptions as outlined in the Methods section. If any of these are wrong, these results are likely biased accordingly. However, every effort was made to be as conservative as possible in making the assumptions, which would likely translate to an underestimation of the true number of malaria deaths prevented from malaria prevention interventions over the past decade. Third, projecting the proportion and number of deaths due to malaria from a baseline year to subsequent years using the LiST model, for both the counterfactual and intervention scale-up, does not account for unmeasured influences that may affect malaria mortality in subsequent years, such as changes in environmental conditions, insecticide resistance, urbanization, overall development or political stability. Fourth, it is highly likely the PE of vector control is heterogeneous across gradients of malaria transmission, by type of vector, child age, and age and integrity of the ITN. However, due to a paucity of available data, it is not currently possible to vary the PE of ITNs by potential effect-modifying variables. However, there is an on-going attempt to refine the PE of ITNs interventions in LiST to be sensitive to these effect modifiers, including a community effect at high coverage levels. It is hoped future estimates will be refined accordingly. Fifth, the PE of IPTp was partially based on data from trials previous to widespread SP resistance; the PE of IPTp with SP may have diminished in the latter years of the past decade and are not accounted for in this analysis. Sixth, modelling case management of malaria with prompt effective treatment with ACT is currently not possible in LiST, primarily due to a lack of data that can be matched between coverage indicators and the PE of ACT [24]. Inclusion of ACT in future modelling efforts would likely increase the number of child deaths prevented.

As previously mentioned, two studies lend credibility to the LIST model's ability to retrospectively estimate the effect of ITNs preventing child malaria deaths. First, the LiST model did reasonably well compared to estimates of measured reductions in < 5 mortality following the scale-up of ITNs in 4 studies across a range of transmission setting [40]. Second the effectiveness of household ownership of ≥ 1 ITN at preventing all-cause post-neonatal mortality under routine malaria program settings was recently found to have nearly the identical effectiveness as that seen in the ITNs trials, upon which the LiST model ITN PE is based [7].

The estimated prevention of malaria child deaths over the past decade may appear to show only a moderate impact on child mortality. In fact these projections are very conservative. First, this analysis did not include the deaths prevented from prompt treatment of fevers with ACT. By 2008, all but one malaria-endemic country in Africa had adopted ACT as the first-line drug for uncomplicated *Plasmodium falciparum *malaria [9]. This increased access to effective anti-malarials is very likely to have prevented an additional number of malaria deaths over the past decade. However insufficient data on testing and treatment precluded this from being part of these analyses. Second, this analysis does not account for the likely benefit of ITN scale-up on indirect malaria mortality where malaria is prevented as a co-infection that contributes to a child death [41]. Third, the effect of malaria prevention in pregnancy acts solely through IUGR in LiST and not specifically through prematurity, which would likely have a larger impact on neonatal mortality [30]. Fourth, ITN coverage of pregnant women was estimated by ITN use by pregnant women the previous night to be most conservative, which likely provides an underestimation of the true protection conferred to women as a result of the community effect from vector control interventions. Fifth, this analysis defaulted to the most conservative estimates of yearly intervention coverage changes where interpolations were made between survey estimates. Finally, IRS, which is estimated to have an effect on malaria-caused mortality similar to that of ITNs, was not included in this analysis because of the paucity of national level coverage estimates 2001-2010.

These modelling results suggest that funding for malaria prevention in Africa over the past decade has had a substantial effect on decreasing child malaria deaths. Declines in child malaria deaths as a result of achieving universal coverage of malaria prevention interventions, especially in combination with improved access to diagnosis and effective case management, will likely contribute substantially to meeting MDG 4, to reduce the < 5 mortality rate by two thirds by 2015. Moreover, most African countries will be able to meet the MDG 6 (Target 3) of halting and reversing trends in malaria incidence with successful scale-up of malaria control interventions. However, it has been estimated that a substantial funding gap remains to achieve full malaria control scale-up in Africa; international donors must do more to allow countries to achieve and maintain universal coverage as rapidly as possible [36,60,61].

## Competing interests

The authors have declared there to be no financial or non-financial competing interests.

## Authors' contributions

T Eisele coordinated the analyses and prepared all drafts of the paper, incorporating comments by co-authors. D Larsen led the analysis of all data using the LiST model. R Cibulskis, J Yukich and C Zikusooka led the cost-effective analysis. All named authors contributed to the conceptualization, analysis and/or completion of the estimates, as well as editing of the paper.

## Financial disclosure

This work was funded in part by a grant to the US Fund for UNICEF from the Bill & Melinda Gates Foundation (grant 43386) and from a grant to the Malaria Control and Evaluation Partnership in Africa (MACEPA)- a programme at PATH- from the Bill & Malinda Gates Foundation. No funding bodies had any role in study design, data collection and analysis, decision to publish, or preparation of the manuscript.

## Supplementary Material

Additional file 1 **Expansion of methods for the Lives Saved Tool analysis**. Appendix of methods expanding on the methods used in this analysis. Click here for file

Additional file 2 **Tables of estimated lives saved by country and year**. Country specific estimates of child deaths prevented by ITNs and by malaria prevention in pregnancy interventions. Click here for file

Additional file 3 **Figures of malaria prevention coverage estimates 2001-2010 for each country**. Country specific estimates of malaria prevention in pregnancy (IPTp/ITNs) coverage in rural areas from 2000-2010. Click here for file

Additional file 4 **Table of malaria in pregnancy intervention coverage estimates from household surveys 2000-2010**. Table of malaria in pregnancy intervention coverage estimates from household surveys 2000-2010. Click here for file

Additional file 5 **Tables and additional details of the cost-effectiveness methods and results of sensitivity analysis**. Tables and additional details of the cost-effectiveness methods and results of sensitivity analysis. Click here for file
