# Supplementary material for: Estimates of child deaths prevented from malaria prevention scale-up in Africa 2001-2010
Source: Malar J. 2012 Mar 28;11:93. doi: 10.1186/1475-2875-11-93 (PMC3350413; doi:10.1186/1475-2875-11-93)
Supplement: Additional file 1 — Expansion of methods for the Lives Saved Tool analysis. Appendix of methods expanding on the methods used in this analysis. [file 1475-2875-11-93-S1.DOC]

**Additional file 1: Expansion of methods for estimating malaria deaths prevented 2001-2010 using the Lives Saved Tool (LiST) model**

1. **African countries included in this analysis**

Forty-three countries in malaria-endemic Africa were included in the analysis of the impact of vector control on child mortality (Figure 1). Cape Verde, Comoros and Lesotho were excluded due to the low number of malaria deaths in these countries . The 36 countries included in the analysis represent 99% of the population at risk of malaria in sub-Saharan Africa (SSA) or 99% of the malaria-caused mortalities in 2000 in SSA .

Thirty-two malaria endemic countries were included in the analysis of the impact of malaria prevention in pregnancy on malaria-caused child mortality. Cape Verde, Comoros, Eritrea, Burundi, Ethiopia, Mauritania, Botswana, Djibouti, Namibia, Sao Tome and Principe, South Africa, Lesotho, and Swaziland were excluded because malaria prevention in pregnancy has little to no effect in countries with low transmission, thus there is no official policy for malaria prevention in pregnancy with intermittent preventive treatment (IPTp) in these countries . The 32 countries included in the analysis account for 88% of the population in SSA at risk of malaria and 90% of the malaria-caused child deaths in 2000 in SSA .

1. **LiST model overview**

The LiST model used in this analysis (version 4.22) and accompanying documentation can be downloaded from [www.jhsph.edu/dept/ih/IIP/list/](http://www.jhsph.edu/dept/ih/IIP/list/). LiST is a computer projection model used to estimate the number of deaths that can be prevented as a result of scaling up effective child health interventions. A complete description of the uses of LiST and background on its creation, including expert technical inputs, are described in detail elsewhere . LiST is programmed as a module in the demographic projection model SPECTRUM, as described elsewhere . LiST uses a simple cohort model that follows children through five age bands from birth to five years to estimate the number of neonatal and child deaths that could be prevented by different intervention scale-up scenarios.

The model can be used to make future projections of deaths prevented from intervention scale-up, compared to a baseline of the current year, or can be used retrospectively to estimate the number of deaths that were prevented in the past from intervention scale-up, compared to a historical baseline year. The model estimates child deaths prevented (within specific cause of death categories) due to intervention scale-up within a specified country as a function of three primary parameters: 1) the number of child deaths by cause projected to occur in each year (including population growth parameters over time); 2) the protective effect (PE) on cause-specific mortality (PE = 1-relative risk*100) for each intervention being scaled-up; and 3) increases in population coverage of each intervention. After accounting for population growth, the model computes the number of deaths prevented by cause each year as the difference between the estimated deaths that occur with intervention scale-up and the estimated deaths that would have occurred had no scale-up occurred beyond the level at a baseline year. The following basic equation is used within the model to estimate the number of child malaria deaths 1-59 months that were prevented from increases in vector control coverage (insecticide-treated mosquito nets [ITNs] and indoor-residual spraying [IRS]), where cause *i* is malaria and intervention *j* is vector control:

Deaths Averted^ij^_s_ = Deaths Averted Total_s_ * (%RedMort_t_^ij^ / %RedMort_s_^Total^)

%RedMort^ij^_s_ = [I^ij^ * (P^j^_t_ – P^j^_0_) ]/ (1 - I^ij^* P^j^_0_), where

%RedMort^ij^_s_ = % reduction in mortality from cause i by scale-up of intervention j

I^ij^ = effectiveness of intervention j in reducing mortality from cause i

P^j^_0_ = baseline coverage of the intervention j

P^j^_s_ = scale-up coverage for the intervention j

While low birth weight (LBW) is due to either intrauterine growth retardation (IUGR) or preterm delivery, the effect of malaria prevention interventions during pregnancy (either through ITNs or IPTp) on LBW in the LiST model acts solely through IUGR. The effect of reducing IUGR has two effects in the LiST model for estimating <5 child deaths prevented, as noted elsewhere .  First, children with IUGR have a greater relative risk (RR) of dying during the neonatal period, with increased RR of dying due to diarrhea [RR = 2.0], sepsis/pneumonia (RR =2.0), and asphyxia (RR = 2.3).  Second, IUGR increases the chance that the child will be stunted, which in turn increases the RR for measles, malaria, diarrhea and pneumonia deaths in the post-neonatal period. In this analysis, the effect of malaria prevention in pregnancy acted only on deaths from the first 2 pregnancies of women in each country. The following basic equation is used within the model to estimate the number of child malaria deaths 1-59 months that can be prevented from malaria prevention in pregnancy, where cause *i* is deaths from IUGR and intervention *j* is composite indicator for ITNs and IPTp in the first 2 pregnancies:

%RedIUGR^j^ = I^j^× (P^j^_t_ – P^j^ _0_) /(1 – I^j^×P^j^ _0_), where

%RedIUGR^j^ = percent of reduction in IUGR due to intervention scale-up of j

I^j^ = proportion by which intervention j reduces IUGR

P^j^_0_ = baseline coverage of the intervention j

P^j^_s_ = scaled-up coverage for the intervention j

1. **Within country estimates of cause-specific child deaths**

Within the LiST model, the number of disease/condition-specific deaths among children under 5 years old in each country is based on country-specific estimates of cause-specific mortality for all low- and middle-income countries, whereby a disease/condition-specific mortality profile is applied to the estimated total number of deaths among children <5 estimated for each country each year. The total number of <5 child deaths for the baseline year of 2000, by age, is based on estimates of under-five mortality produced by the UN Inter-agency Group for Child Mortality Estimation (IGME) . The number of malaria deaths in children 1-59 months in our baseline year of 2000 was estimated as the proportion of the 1-59 month all-cause mortality envelope attributable to malaria by the CHERG for each sub-Saharan African country . Estimates used in the analysis differ slightly than those reported previously by Rowe and colleagues (2006) because of revisions to the all-cause <5 year death envelopes in some countries.

1. **Estimates of intervention effectiveness**

As described in detail elsewhere, the PE of vector control for preventing malaria deaths in children 1-59 months has been estimated to be 55% (range 49% - 60%) based on a systematic review of related trials and studies . The PE of malaria prevention during pregnancy for preventing low-birth weight has been estimated to be 35% (95% confidence interval [CI] 23-45%) during the first 2 pregnancies in malaria endemic areas in the presence of SP resistance based on a systematic review of related trials .

1. **Estimating yearly intervention coverage for malaria prevention in pregnancy (IPTp/ITN) 2000-2010**

As outlined elsewhere, protection by malaria prevention in pregnancy, defined as the higher of each country’s estimates of either proportion of pregnant women using an ITN the previous night or the proportion of women who had a live birth in the past 2 years who received 2+ doses of sulfadoxamine-pyremethamine (SP) during an ante-natal care (ANC) visit (IPTp), were used in the LiST analysis. These estimates were obtained from the final reports of DHS, MICS, MIS and Office of National Statistics (ONS) surveys (Additional file 2). If the survey did not specify the number of doses of SP or where it was received, the estimate of IPTp taken during pregnancy without specification of number of doses or if taken at ANC was used. This occurred in the following surveys: Congo 2005, Liberia 2005, and Malawi 2000.

Standard errors were ascertained from survey datasets to obtain 95% CIs about survey point estimates. In the case that standard errors about the estimate were unavailable, the standard errors were imputed using formula 1.

The year prior to the country declaring IPTp with SP as national policy was set to 0%, for those countries using IPTp as the coverage indicator. It was unclear what year Liberia adopted the policy, and so the year 2000 was set to 0 in this case. Where the higher indicator for malaria prevention in pregnancy was ITN use by pregnant women, the coverage of ITNs among pregnant women was set to 0% in 2000. This may lead to a slight overestimation of the impact of malaria prevention in pregnancy as pregnant women may have been using ITNs in the year 2000. However the overall contribution of malaria prevention in pregnancy to the total number of lives saved is quite small.

Linear interpolation was used between the first year of measured coverage and the next available survey point estimate. Linear interpolation was also used between multiple surveys where available. The slope between the most recent household survey and the earliest household survey was used to inform the increase for years beyond the most recent household survey through 2010. In the case that there was only 1 household survey in the country, the year set to 0 was used in place of the earliest household survey. Coverage was assumed to never decrease unless surveys estimated otherwise.

Resultant IPTp/ITN coverage changes for each country 2000-2010, with uncertainty, are presented in Figure 3.

1. **Cost and Cost Effectiveness**

A review of existing literature on the costs and cost effectiveness of ITN delivery [ITN or long-lasting ITN (LLIN)] in Africa since 2005 was conducted in PubMed and the grey literature. The systematic review identified 35 studies. Of these, 13 contained data in sufficient detail for the estimation of ITN delivery costs . Costs of ITN programs were separated into delivery and commodity components, and used to estimate a median cost of ITN delivery, and of ITNs or LLINs procured for programs. The cost of an ITN or LLIN itself was estimated based on the literature. The median cost of the ITN itself was then annualized according to methodology in previous studies and guidelines. All costs were adjusted to 2009 USD using the US GDP deflator and discounted to reflect the present values at the start of simulations (United States Bureau of Economic Analysis: [http://www.bea.gov](http://www.bea.gov/)). The cost of 1 year of ITN exposure was applied to the each year of ITN availability. The number of LLINs delivered from manufacturers to all sub-Saharan African countries was available from the Alliance for Malaria Prevention Net Mapping Project for the period 2004-2009 and was used to estimate the number of available ITNs in all relevant sub-Saharan African countries in each year from 2006 to 2009 assuming that nets lasted for a three year period . The following LLIN manufacturers were represented by the AMP data: Sumitomo/A-Z, Vestergaard-Frandsen, Clarke, BASF, Intection, Tana Netting, and Yorkool. Lives saved over this time period were estimated using the LiST model as described previously and Disability Adjusted Life Years were calculated using a generic sub-Saharan African life table, assuming that all lives saved resulted in a gain of 25 discounted DALYs. Both costs and effects used a 3% discount rate as is typical of health economic literature. Because cost-effectiveness results may be potentially biased by errors in cost or effects measurement as well as other assumptions, a one-way sensitivity analysis was conducted focusing on costs of ITNs, estimates of lives saved, and discount rate. ITN delivery cost, net lifetime, LLIN commodity cost, discount rate, and the effectiveness of the intervention were varied in the sensitivity analysis.

We estimated the median cost of delivery of an ITN to be US$ 1.64 (Additional file 5, Table 1). There was some difference between subgroups of delivery systems (median US$ 1.34 for mass campaigns and US$ 3.96 for retail distribution). We estimated the median cost of an ITN to be US$ 5.44. When discounted over a three year period, one year of ITN exposure was valued at US$ 1.85.

The results of sensitivity analysis are presented in Additional file 5, Table 2. The results were most sensitive to the cost of delivery and the LLIN itself and the effectiveness of the net, however, within the parameters of the high and low bounds of effectiveness derived from the LiST model and the costs derived from the literature review the intervention remained very cost-effective under all sensitivity scenarios. Additionally numbers were calculated using only cost studies which referred to mass campaigns or other delivery systems published since 2006, where nets were mainly LLINs. The result did not change substantially.

1. **Uncertainty estimation of LiST estimates**

Uncertainty bounds about total estimated malaria deaths prevented from vector control 2001-2010 were estimated by projecting lives saved when varying the three primary model parameters: 1) estimated malaria deaths within each country; 2) the estimated PE of vector control on malaria mortality; and 3) intervention coverage changes 2000-2010. Using this approach, lower and upper uncertainty bounds were estimated using a high impact / low impact scenario. The uncertainty about the number of malaria deaths among children 1-59 months was derived from the 95% confidence intervals about the proportion of all deaths due to malaria in this age group in 2000 estimated by the CHERG . The reported range of 49-60% about the 55% PE of vector control for preventing malaria mortality was used as the uncertainty about this parameter in this analysis . The uncertainty about ITN scale-up is dependent on the percent change, or the slope of the vector control coverage curve, from 2000-2010. Under the largest-increase scenario, the percent change in coverage was set to that from the lower bound of the 95% CI in 2000 to the upper bound in 2010, resulting in the greatest slope during this period. Under the smallest-increase scenario, the percent change in coverage was set to that from the upper bound of the 95% CI in 2000 to the lower bound in 2010, resulting in the least slope during this period. The slope of each uncertainty bound between 2000-2010 was then calculated in the same manner as the slope of the midpoint over this period (see Figure 1 for resultant uncertainty of vector control scale-up 2000-2010).

Uncertainty bounds about total estimated child deaths prevented from malaria prevention during pregnancy 2001-2010 were estimated by projecting lives saved while two primary model parameters: 1) the estimated PE of malaria prevention in pregnancy on preventing LBW; and 2) intervention coverage. The 95% CI of 23-45% about the 35% PE of malaria prevention in pregnancy for preventing LBW was used as the uncertainty about this parameter in this analysis . The uncertainty about IPTp/ITN scale-up under the largest-increase / smallest-increase scenario followed the methodology for vector control outlined above. Linear interpolation was used between bounds (see Figure 2 for resultant uncertainty of malaria prevention in pregnancy scale-up 2000-2010).

1. **Estimating total continental coverage**

Yearly coverage estimates for each country were weighted according to each country’s 2005 population estimate (mid-point of 2000-2010) to estimate the continental coverage rates of vector control in rural areas and malaria in pregnancy prevention in rural areas.

1. **Coverage estimation of non-malaria child survival interventions in this analysis**

In LiST, changing coverage of non-malaria child survival interventions affect the overall <5 child mortality envelope, and thereby may affect the malaria-specific child mortality envelope over time, which is the product of the all-cause envelope X proportion of all-cause child deaths due to malaria. This analysis accounted for the scale-up of non-malaria child survival interventions, such as vaccinations and antenatal care, accordingly. Coverage estimates for these interventions were derived from nationally representative household surveys; years without a survey point estimate were kept constant at the value of the last available survey estimate (e.g. if there were surveys in 2003 and 2007 for a particular country, values for 2004, 2005 and 2006 were set to that of 2003, and values for 2008, 2009 and 2010 were set to that of 2007).

1. **Coverage estimation of prompt treatment of fevers with ACTs in this analysis**

The coverage of case management of malaria with ACTs was held constant in this analysis. As LiST is driven by a change in coverage, these indicators were then assumed to have no effect on child mortality throughout the time period.

**References**

1. Rowe AK, Rowe SY, Snow R, Korenromp EL, Armstrong-Schellenberg JR, Stein C, Nahlen BL, Bryce J, Black RE, Steketee R: Estimates of the burden of mortality directly attributable to malaria for children under 5 years of age in Africa for the year 2000 Final report. For the Child Health Epidemiology Reference Group (CHERG). In*.* Washington DC: Child Health Epidemiology Reference Group (CHERG); 2006.

2. Hay SI, Guerra CA, Gething PW, Patil AP, Tatem AJ, Noor AM, Kabaria CW, Manh BH, Elyazar IR, Brooker S, Smith DL, Moyeed RA, Snow RW: A world malaria map: Plasmodium falciparum endemicity in 2007. *PLoS Med* 2009, 6:e1000048.

3. Steketee RW, Nahlen BL, Parise ME, Menendez C: The burden of malaria in pregnancy in malaria-endemic areas. *Am J Trop Med Hyg* 2001, 64:28-35.

4. WHO: World Malaria Report 2009. In*.* Edited by WHO. Geneva: WHO; 2009.

5. Boschi-Pinto C, Young M, Black RE: The Child Health Epidemiology Reference Group reviews of the effectiveness of interventions to reduce maternal, neonatal and child mortality. *Int J Epidemiol* 2010, 39 Suppl 1:i3-6.

6. Stover J, McKinnon R, Winfrey B: Spectrum: a model platform for linking maternal and child survival interventions with AIDS, family planning and demographic projections. *Int J Epidemiol* 2010, 39 Suppl 1:i7-10.

7. Bhutta ZA, Ahmed T, Black RE, Cousens S, Dewey K, Giugliani E, Haider BA, Kirkwood B, Morris SS, Sachdev HP, Shekar M: What works? Interventions for maternal and child undernutrition and survival. *Lancet* 2008, 371:417-440.

8. Friberg IK, Bhutta ZA, Darmstadt GL, Bang A, Cousens S, Baqui AH, Kumar V, Walker N, Lawn JE: Comparing modelled predictions of neonatal mortality impacts using LiST with observed results of community-based intervention trials in South Asia. *Int J Epidemiol* 2010, 39 Suppl 1:i11-20.

9. UNICEF: Levels and Trends of Child Mortality in 2006: Estimates developed by the Inter-agency Group for Child Mortality Estimation. In*.* New York: UNICEF, WHO, The World Bank and UN Population Division; 2007.

10. Rowe AK, Rowe S, Snow RW, Korenromp E, Armstrong Schellenberg J, Stein C, Nahlen BL, Bryce J, Black RE, Steketee RW: The burden of malaria mortality among African children in the year 2000. *Int J Epidemiol* 2006, 35:691-704.

11. Eisele TP, Larsen D, Steketee RW: Protective efficacy of interventions for preventing malaria mortality in children in Plasmodium falciparum endemic areas. *Int J Epidemiol* 2010, 39:i88-101.

12. WHO: World Malaria Report 2005. In*.*: World Health Organization; 2005.

13. WHO: World Malaria Report 2008. In*.* Edited by WHO. Geneva: WHO; 2008.

14. Grabowsky M, Nobiya T, Ahun M, Donna R, Lengor M, Zimmerman D, Ladd H, Hoekstra E, Bello A, Baffoe-Wilmot A, Amofah G: Distributing insecticide-treated bednets during measles vaccination: a low-cost means of achieving high and equitable coverage. *Bull World Health Organ* 2005, 83:195-201.

15. Kolaczinski JH, Kolaczinski K, Kyabayinze D, Strachan D, Temperley M, Wijayanandana N, Kilian A: Costs and effects of two public sector delivery channels for long-lasting insecticidal nets in Uganda. *Malar J* 2010, 9:102.

16. Mueller DH, Wiseman V, Bakusa D, Morgah K, Dare A, Tchamdja P: Cost-effectiveness analysis of insecticide-treated net distribution as part of the Togo Integrated Child Health Campaign. *Malar J* 2008, 7:73.

17. Mulligan J, Yukich J, Hanson K: Costs and effects of the Tanzanian national voucher scheme for insecticide-treated nets. *Malar J* 2008, 7:32.

18. Stevens W, Wiseman V, Ortiz J, Chavasse D: The costs and effects of a nationwide insecticide-treated net programme: the case of Malawi. *Malar J* 2005, 4:22.

19. Yukich JO, Lengeler C, Tediosi F, Brown N, Mulligan JA, Chavasse D, Stevens W, Justino J, Conteh L, Maharaj R, Erskine M, Mueller DH, Wiseman V, Ghebremeskel T, Zerom M, Goodman C, McGuire D, Urrutia JM, Sakho F, Hanson K, Sharp B: Costs and consequences of large-scale vector control for malaria. *Malar J* 2008, 7:258.

20. Yukich JO, Zerom M, Ghebremeskel T, Tediosi F, Lengeler C: Costs and cost-effectiveness of vector control in Eritrea using insecticide-treated bed nets. *Malar J* 2009, 8:51.

21. De Allegri M, Marschall P, Flessa S, Tiendrebeogo J, Kouyate B, Jahn A, Muller O: Comparative cost analysis of insecticide-treated net delivery strategies: sales supported by social marketing and free distribution through antenatal care. *Health Policy Plan* 2010, 25:28-38.

22. Becker-Dreps SI, Biddle AK, Pettifor A, Musuamba G, Imbie DN, Meshnick S, Behets F: Cost-effectiveness of adding bed net distribution for malaria prevention to antenatal services in Kinshasa, Democratic Republic of the Congo. *Am J Trop Med Hyg* 2009, 81:496-502.

23. WHO: Costing of the Zanzibar Malaria Control Program ITN program: Report of a concultation 2009. In*.* Edited by WHO. Geneva; 2009.

24. WHO: Costing of the Uganda ITN activities: Report of a concultation 2009. In*.* Edited by WHO. Geneva; 2009.

25. WHO: Costing of PSI Kenya ITN program: Report of a concultation draft 3 2009. In*.* Edited by WHO. Geneva; 2009.

26. Bonner K, Mwita A, McElroy PD, Omari S, Mzava A, Lengeler C, Kaspar N, Nathan R, Ngegba J, Mtung'e R, Brown N: Design, implementation and evaluation of a national campaign to distribute nine million free LLINs to children under five years of age in Tanzania. *Malar J* 2011, 10:73.

27. Drummond MF, Sculper MJ, Torrence GW, O'Brien BJ, Stoddart GL: Methods for the Economic Evaluation of Health Care Programmes, 3rd edn. Oxford: Oxford University Press; 2005.

28. Baltussen R, Adams T, Tan Torres T, Hutubessy R, Acharya A, Evans DB, Murray CJL: Making Choices in Health: WHO Guide to Cost-Effectiveness Analysis. Geneva: WHO; 2003.

29. Kilian A, Byamukama W, Pigeon O, Atieli F, Duchon S, Phan C: Long-term field performance of a polyester-based long-lasting insecticidal mosquito net in rural Uganda. *Malar J* 2008, 7:49.

30. Erlanger TE, Enayati AA, Hemingway J, Mshinda H, Tami A, Lengeler C: Field issues related to effectiveness of insecticide-treated nets in Tanzania. *Med Vet Entomol* 2004, 18:153-160.

31. Drummond M, Manca A, Sculpher M: Increasing the generalizability of economic evaluations: recommendations for the design, analysis, and reporting of studies. *Int J Technol Assess Health Care* 2005, 21:165-171.

32. U.N.: The World Population Prospect: The 2008 Revision. In*.* New York: UN Population Division; 2008.
