# Supplementary material for: Estimates of child deaths prevented from malaria prevention scale-up in Africa 2001-2010
Source: Malar J. 2012 Mar 28;11:93. doi: 10.1186/1475-2875-11-93 (PMC3350413; doi:10.1186/1475-2875-11-93)
Supplement: Additional file 2 — Tables of estimated lives saved by country and year. Country specific estimates of child deaths prevented by ITNs and by malaria prevention in pregnancy interventions. [file 1475-2875-11-93-S2.DOC]

**Additional file 2: Tables of estimated lives saved by country and year**

**Table 1: Estimated malaria deaths in children one-59 months prevented by vector control scale-up 2001-2010**

| **Country** | **Malaria deaths 2000*** | **2001** | **2002** | **2003** | **2004** | **2005** | **2006** | **2007** | **2008** | **2009** | **2010** | **Total** | **Uncertainty** | |
| --- | --- | --- | --- | --- | --- | --- | --- | --- | --- | --- | --- | --- | --- | --- |
|  |  |  |  |  |  |  |  |  |  |  |  |  | Lower Bound | Upper Bound |
| Angola | 13783 | 130 | 148 | 103 | 61 | 671 | 1721 | 2002 | 1866 | 2225 | 2837 | 11,764 | 7210 | 26258 |
| Benin | 10884 | 67 | 98 | 108 | 160 | 590 | 1855 | 3264 | 4062 | 41111 | 3821 | 18,136 | 8092 | 31609 |
| Botswana | 4 | 0 | 0 | 0 | 0 | 0 | 1 | 1 | 1 | 1 | 1 | 5 | 2 | 10 |
| Burkina Faso | 19954 | 94 | 283 | 634 | 786 | 1420 | 2521 | 2834 | 3340 | 5271 | 8056 | 25,239 | 15,608 | 46,243 |
| Burundi | 4285 | 8 | 11 | 17 | 69 | 155 | 283 | 434 | 648 | 1006 | 1464 | 4095 | 2935 | 8962 |
| Cameroon | 21331 | 50 | 56 | 122 | 226 | 1175 | 2745 | 3173 | 3220 | 3608 | 4233 | 18,608 | 7195 | 37145 |
| CAR | 5488 | 14 | 15 | 25 | 66 | 206 | 389 | 469 | 473 | 500 | 557 | 2713 | 1532 | 6380 |
| Chad | 18594 | -38 | -143 | -172 | -290 | -324 | -116 | 175 | 405 | 570 | 753 | 820 | 3048 | 9883 |
| Congo (Brazzaville) | 4863 | 7 | 10 | 18 | 46 | 116 | 172 | 190 | 220 | 269 | 322 | 1370 | 651 | 3844 |
| Cote d’Ivoire | 19209 | 9 | -20 | -26 | -83 | -73 | 290 | 719 | 948 | 1088 | 1222 | 4074 | 2537 | 11,97 |
| DRC | 78901 | 198 | 217 | 156 | 409 | 1248 | 2196 | 5328 | 17960 | 26561 | 26964 | 81231 | 61528 | 124408 |
| Djibouti | 84 | 0 | 0 | 1 | 1 | 2 | 5 | 18 | 48 | 49 | 44 | 168 | 85 | 252 |
| Equatorial Guinea (Bioko Island) | 934 | 1 | 2 | 3 | 4 | 12 | 32 | 172 | 380 | 298 | 189 | 1093 | 691 | 1622 |
| Eritrea | 1475 | 445 | 661 | 834 | 860 | 857 | 703 | 639 | 862 | 722 | 621 | 7204 | 4539 | 9998 |
| Ethiopia | 20,657 | 53 | 114 | 157 | 212 | 476 | 2648 | 7512 | 12,176 | 11,114 | 7588 | 42,050 | 27,247 | 59,401 |
| Gabon | 1520 | 3 | 5 | 10 | 24 | 63 | 171 | 370 | 580 | 521 | 347 | 2094 | 918 | 3901 |
| Gambia | 2101 | -21 | -67 | -91 | -100 | 144 | 166 | -26 | 107 | 278 | 327 | 717 | 1872 | 4817 |
| Ghana | 26808 | 60 | 130 | 285 | 382 | 1032 | 2277 | 4046 | 6024 | 7414 | 7584 | 29,234 | 20405 | 47205 |
| Guinea | 0 | 19 | 26 | 42 | 26 | 29 | 155 | 415 | 720 | 939 | 1068 | 3439 | 1810 | 7922 |
| Guinea-Bissau | 2131 | -15 | -29 | -36 | -43 | 218 | 475 | 482 | 596 | 747 | 762 | 3157 | 2175 | 7373 |
| Kenya | 19937 | 120 | 159 | 239 | 175 | 1752 | 5527 | 7679 | 7921 | 8187 | 8411 | 4,170 | 30749 | 71456 |
| Liberia | 4583 | 8 | 14 | 27 | 53 | 134 | 273 | 413 | 796 | 1296 | 1501 | 4515 | 2833 | 7160 |
| Madagascar | 14235 | 13 | -8 | -46 | 103 | 2188 | 4665 | 5842 | 4924 | 3899 | 5661 | 27241 | 19661 | 39,721 |
| Malawi | 10543 | 87 | 380 | 1322 | 1841 | 1792 | 2413 | 2877 | 2345 | 2101 | 3650 | 18808 | 11487 | 29313 |
| Mali | 21955 | 55 | 119 | 265 | 595 | 2095 | 5540 | 9423 | 11889 | 12050 | 11026 | 53057 | 39411 | 76249 |
| Mauritania | 1212 | 2 | 1 | 0 | 3 | 18 | 42 | 70 | 83 | 82 | 84 | 385 | 223 | 863 |
| Mozambique | 30832 | 130 | 327 | 726 | 674 | 480 | 1103 | 2437 | 4653 | 6407 | 7296 | 24233 | 15491 | 45872 |
| Namibia | 3 | 0 | 0 | 0 | 0 | 0 | 0 | 1 | 1 | 2 | 2 | 6 | 0 |  |
| Niger | 13840 | 41 | 91 | 242 | 574 | 2448 | 5292 | 5333 | 4641 | 6566 | 8856 | 34084 | 26059 | 55228 |
| Nigeria | 335596 | 330 | 399 | 445 | -76 | 72 | 1344 | 8067 | 19125 | 37820 | 96636 | 164162 | 96836 | 238960 |
| Rwanda | 1997 | 4 | 11 | 25 | 57 | 206 | 542 | 719 | 751 | 886 | 1095 | 4296 | 3135 | 6131 |
| STP | 269 | -1 | -16 | -17 | -23 | -12 | 7 | 6 | 3 | 12 | 25 | -16 | -240 | 345 |
| Senegal | 12006 | 52 | 123 | 296 | 706 | 978 | 1703 | 2024 | 2804 | 5131 | 5454 | 19271 | 15040 | 32688 |
| Sierra Leone | 8291 | 0 | -10 | -17 | -6 | 68 | 192 | 781 | 1754 | 1628 | 2740 | 7130 | 5942 | 11,500 |
| Somalia | 2291 | 6 | 9 | 12 | 11 | 28 | 139 | 264 | 318 | 335 | 345 | 1467 | 629 | 2713 |
| South Africa | 59 | 4 | 4 | 4 | 4 | 4 | 4 | 5 | 6 | 8 | 9 | 52 | 21 | 190 |
| Sudan | 35623 | 54 | 106 | 170 | 308 | 1333 | 3785 | 4313 | 3529 | 4144 | 5082 | 22824 | 11153 | 41818 |
| Swaziland | 14 | 0 | 0 | 0 | 0 | 0 | 1 | 1 | 1 | 2 | 3 | 8 | 2 | 19 |
| Tanzania | 41054 | 186 | 442 | 1062 | 3501 | 4640 | 6470 | 10,102 | 8200 | 11058 | 19437 | 65098 | 44996 | 100403 |
| Togo | 5626 | 49 | 136 | 355 | 1469 | 2009 | 1323 | 1418 | 1825 | 2376 | 2306 | 13266 | 9684 | 19769 |
| Uganda | 43517 | 52 | 87 | 153 | 317 | 1233 | 4280 | 6391 | 7846 | 11074 | 13450 | 44883 | 31801 | 69342 |
| Zambia | 14289 | 293 | 482 | 483 | 556 | 1964 | 3507 | 4480 | 5329 | 6364 | 5354 | 28812 | 20739 | 47823 |
| Zimbabwe | 150 | 0 | 0 | 1 | 2 | 6 | 13 | 27 | 44 | 46 | 44 | 183 | 86 | 361 |
| **Total** | **870928** | **2569** | **4373** | **7937** | **13653** | **31453** | **66854** | **104890** | **143424** | **188766** | **267227** | **831146** | **562777** | **1364645** |

*Estimates of malaria deaths in 2000 differ slightly from those published previously by Rowe and colleagues 2006 [25] due to revisions of estimates of the overall all-cause child mortality envelope for each country.

Vector control: Houses protected by either ITNs and/or IRS

CAR: Central African Republic

DRC: Democratic Republic of Congo

STP: Sao Tome and Principe

Table 2: Estimated <5 child deaths prevented by malaria prevention in pregnancy scale-up 2001-2010

| **Country** | **Neonatal deaths in 2000** | **2001** | **2002** | **2003** | **2004** | **2005** | **2006** | **2007** | **2008** | **2009** | **2010** | **Total** | **Uncertainty** | |
| --- | --- | --- | --- | --- | --- | --- | --- | --- | --- | --- | --- | --- | --- | --- |
|  |  |  |  |  |  |  |  |  |  |  |  |  | Lower Bound | Upper Bound |
| Angola | 31951 | 11 | 22 | 34 | 47 | 62 | 76 | 90 | 107 | 125 | 144 | 718 | 342 | 1075 |
| Benin | 9132 | 0 | 1 | 1 | 1 | 2 | 2 | 2 | 3 | 3 | 4 | 19 | 9 | 31 |
| Burkina Faso | 18,776 | 0 | 0 | 0 | 0 | 0 | 1 | 1 | 2 | 2 | 3 | 9 | 1 | 33 |
| Cameroon | 17,463 | 0 | 0 | 0 | 3 | 8 | 13 | 18 | 23 | 29 | 35 | 129 | 56 | 227 |
| CAR | 7450 | 0 | 0 | 0 | 1 | 2 | 4 | 5 | 6 | 8 | 9 | 35 | 16 | 60 |
| Chad | 18,779 | 0 | 0 | 0 | 3 | 7 | 11 | 15 | 20 | 25 | 30 | 111 | 62 | 171 |
| Congo (Brazzaville) | 3496 | 0 | 0 | 0 | 0 | 1 | 1 | 2 | 2 | 3 | 4 | 13 | 1 | 28 |
| Cote d’Ivoire | 25,092 | 0 | 0 | 0 | 0 | 9 | 18 | 29 | 39 | 51 | 62 | 208 | 105 | 330 |
| DRC | 123,220 | 0 | 0 | 0 | 6 | 14 | 21 | 29 | 109 | 201 | 298 | 678 | 381 | 1013 |
| Equatorial Guinea  (Bioko Island) | 1169 | 0 | 0 | 0 | 0 | 1 | 1 | 2 | 3 | 4 | 5 | 16 | 7 | 22 |
| Gambia | 2503 | 0 | 1 | 3 | 4 | 5 | 7 | 9 | 10 | 13 | 15 | 67 | 40 | 89 |
| Ghana | 22,418 | 0 | 0 | 1 | 14 | 27 | 41 | 56 | 72 | 89 | 106 | 406 | 236 | 568 |
| Guinea | 14,668 | 0 | 0 | 0 | 0 | 1 | 3 | 4 | 6 | 8 | 9 | 31 | 9 | 59 |
| Guinea-Bissau | 2524 | 0 | 0 | 0 | 0 | 1 | 3 | 5 | 7 | 9 | 12 | 37 | 20 | 54 |
| Kenya | 32,629 | 2 | 3 | 5 | 7 | 10 | 12 | 15 | 38 | 61 | 72 | 225 | 171 | 343 |
| Liberia | 5919 | 1 | 1 | 2 | 3 | 4 | 11 | 20 | 30 | 41 | 51 | 164 | 88 | 198 |
| Madagascar | 18,524 | 14 | 28 | 35 | 47 | 59 | 71 | 84 | 97 | 111 | 124 | 670 | 396 | 947 |
| Malawi | 13,359 | 7 | 14 | 24 | 31 | 37 | 42 | 47 | 53 | 57 | 61 | 373 | 581 | 693 |
| Mali | 32,295 | 4 | 8 | 12 | 17 | 22 | 27 | 70 | 116 | 167 | 221 | 664 | 377 | 969 |
| Mozambique | 49,251 | 0 | 0 | 0 | 0 | 0 | 40 | 80 | 122 | 165 | 208 | 615 | 0 | 922 |
| Niger | 19,853 | 2 | 4 | 7 | 9 | 12 | 15 | 67 | 126 | 191 | 263 | 696 | 446 | 917 |
| Nigeria | 188,932 | 0 | 2 | 4 | 16 | 30 | 46 | 64 | 83 | 435 | 846 | 1526 | 768 | 2176 |
| Rwanda | 15,355 | 1 | 2 | 2 | 3 | 5 | 11 | 18 | 22 | 26 | 29 | 119 | 70 | 170 |
| Senegal | 14,455 | 0 | 0 | 0 | 6 | 12 | 57 | 65 | 73 | 95 | 118 | 426 | 265 | 573 |
| Sierra Leone | 11,971 | 0 | 0 | 0 | 0 | 1 | 1 | 2 | 3 | 4 | 6 | 17 | 212 | 502 |
| Somalia | 15568 | 0 | 0 | 0 | 0 | 1 | 1 | 2 | 3 | 4 | 5 | 16 | 3 | 24 |
| Sudan | 48404 | 17 | 33 | 51 | 68 | 87 | 105 | 124 | 143 | 163 | 183 | 974 | 315 | 1398 |
| Tanzania | 39624 | 14 | 29 | 47 | 50 | 60 | 71 | 82 | 116 | 152 | 189 | 810 | 473 | 1138 |
| Togo | 7537 | 0 | 0 | 0 | 0 | 4 | 9 | 14 | 19 | 24 | 29 | 99 | 53 | 152 |
| Uganda | 33991 | 6 | 12 | 19 | 27 | 35 | 45 | 72 | 102 | 134 | 155 | 607 | 333 | 947 |
| Zambia | 14579 | 20 | 40 | 64 | 88 | 113 | 140 | 163 | 168 | 184 | 198 | 1178 | 697 | 1621 |
| Zimbabwe | 9043 | 0 | 0 | 0 | 1 | 2 | 3 | 4 | 5 | 6 | 7 | 28 | 12 | 49 |
| **Total** | **1034828** | **99** | **200** | **311** | **452** | **634** | **910** | **1261** | **1729** | **2591** | **3502** | **11689** | **6959** | **17484** |

Malaria prevention in pregnancy: Woman received IPTp during last two pregnancies or currently pregnant women used ITNs previous night

CAR: Central African Republic

DRC: Democratic Republic of Congo

Countries without stable malaria transmission or a policy of IPTp were excluded
