# Supplementary material for: Estimates of child deaths prevented from malaria prevention scale-up in Africa 2001-2010
Source: Malar J. 2012 Mar 28;11:93. doi: 10.1186/1475-2875-11-93 (PMC3350413; doi:10.1186/1475-2875-11-93)
Supplement: Additional file 3 — Figures of malaria prevention coverage estimates 2001-2010 for each country. Country specific estimates of malaria prevention in pregnancy (IPTp/ITNs) coverage in rural areas from 2000-2010. [file 1475-2875-11-93-S3.DOC]

**Additional file 3: Table of** malaria in pregnancy intervention coverage estimates from household surveys 2000-2010

| **Country** | **Year** | **Rural HH estimate** | **Sample Size** | **Rural HH standard error** | **95% confidence interval** | **Survey** |
| --- | --- | --- | --- | --- | --- | --- |
| Angola | 2006 | 26.4^1^ | 269 | 1.6 | 23.3 – 29.5 | MIS |
| Benin | 2006 | 2.3^1^ | 1962 | 0.2 | 1.9 – 2.7 | DHS |
| Burkina Faso | 2006 | 0.3 | 156 | 0.2 | 0.0 – 0.7 | MICS |
| Cameroon | 2006 | 8.9 | 685 | 1.3 | 6.4 – 11.5 | MICS |
| CAR | 2006 | 4.7 | 2510 | 0.6^2^ | 3.5 – 5.9 | MICS |
| Chad | 2010 | 17.7 | 1616 | 1.2 | 15.4 – 20.0 | MICS |
| Congo (Brazzaville) | 2005 | 3.2^3^ | 933 | 0.9^2^ | 1.5 – 4.9 | DHS |
| Cote d’Ivoire | 2006 | 7.2 | 2258 | 0.7 | 5.9 – 8.5 | MICS |
| DRC | 2007 | 4.4 | 2130 | 0.8 | 2.9 – 5.9 | DHS |
| DRC | 2010 | 42.5^1^ | 1024 | 2.6 | 37.2 – 47.7 | MICS |
| Equatorial Guinea^4^ | 2008 | 19.0 | 503 | 2.6 | 16.0 – 24.0 | [36] |
| Gambia | 2006 | 33.8 | 2052 | 1.1 | 31.8 – 35.9 | MICS |
| Ghana | 2003 | 0.9 | 1076 | 0.3 | 0.3 – 1.5 | DHS |
| Gambia | 2010 | 67.2 | 2760 | 1.2 | 64.8 – 69.6 | MICS |
| Ghana | 2006 | 26.1 | 944 | 1.6 | 22.9 – 29.3 | MICS |
| Ghana | 2008 | 43.5 | 811 | 2.5 | 38.6 – 48.3 | DHS |
| Guinea | 2005 | 1.2 | 2122 | 0.3 | 0.7 – 1.7 | DHS |
| Guinea | 2007 | 3.3 | 1669 | 0.9^2^ | 1.6 – 5.0 | ONS |
| Guinea-Bissau | 2006 | 7.1 | 531 | 0.7 | 5.8 – 8.4 | MICS |
| Guinea-Bissau | 2010 | 29.3^1^ | 540 | 2.5 | 24.3 – 34.3 | MICS |
| Kenya | 2003 | 4.4 | 1802 | 0.6 | 3.3 – 5.5 | DHS |
| Kenya | 2007 | 12.8 | 1644 | 1.0^2^ | 10.4 – 14.4 | MIS |
| Liberia | 2005 | 4.3^3^ | 510 | 1.0^2^ | 10.4 – 14.4 | MIS |
| Liberia | 2009 | 44.8 | 1009 | 2.9 | 39.0 – 50.5 | MIS |
| Malawi | 2000 | 28.3 | 4180 | 0.9 | 26.6 – 30.0 | DHS |
| Malawi | 2004 | 42.5^3^ | 4246 | 1.0 | 40.5 – 44.4 | DHS |
| Malawi | 2006 | 45.7 | 9279 | 0.7 | 44.3 – 47.1 | MICS |
| Malawi | 2010 | 50.7 | 213 | 6.9^2^ | 37.3 – 64.1 | MIS |
| Mali | 2006 | 5.6^1^ | 1896 | 0.5 | 4.5 – 6.6 | DHS |
| Mali | 2010 | 41.9 | 1986 | 2.2 | 37.6 – 46.2 | MICS |
| Mozambique | 2007 | 16.1 | 1016 | 1.3^2^ | 13.6 – 18.6 | MIS |
| Niger | 2006 | 4.9 | 1311 | 0.3 | 4.4 – 5.4 | DHS |
| Nigeria | 2003 | 0.2 | 1544 | 0.1 | 0.0 – 0.4 | DHS |
| Nigeria | 2008 | 3.7 | 8311 | 0.3 | 3.1 – 4.3 | DHS |
| Nigeria | 2010 | 38.7 | 586 | 4.0 | 30.8 – 46.6 | MIS |
| Rwanda | 2005 | 15.5 | 776 | 1.0 | 13.4 – 17.6 | DHS |
| Rwanda | 2007 | 59.9 | 580 | 3.0 | 54.1 – 65.7 | DHS |
| Senegal | 2005 | 10.0 | 3126 | 0.8 | 8.4 – 11.7 | DHS |
| Senegal | 2006 | 46.5 | 1352 | 2.3 | 41.9 – 51.1 | MIS |
| Senegal | 2008 | 54.2 | 4450 | 1.4 | 51.3 – 57.0 | MIS |
| Sierra Leone | 2005 | 1.1 | 103 | 1.0^2^ | 0.0 – 3.0 | MICS |
| Sierra Leone | 2008 | 29.3^1^ | 238 | 1.3 | 26.8 – 31.8 | DHS |
| Somalia | 2006 | 0.7 | 1426 | 0.2 | 0.3 – 1.1 | MICS |
| Sudan (Southern) | 2009 | 31.6 | 421 | 4.5^2^ | 22.7 – 40.5 | MIS |
| Tanzania | 2004 | 18.6 | 1908 | 1.0 | 16.6 – 20.7 | DHS |
| Tanzania | 2007 | 27.9 | 2550 | 1.5 | 25.0 – 30.8 | AIS |
| Tanzania | 2010 | 59.2^1^ | 788 | 2.5 | 54.3 – 64.0 | MIS |
| Togo | 2006 | 18.5 | 1213 | 1.3 | 15.9 – 21.1 | MICS |
| Uganda | 2006 | 16.2 | 2956 | 0.9 | 14.4 – 18.0 | DHS |
| Uganda | 2009 | 43.6^1^ | 368 | 4.0 | 36.0 – 51.6 | MIS |
| Zambia | 2006 | 52.5 | 1030 | 1.9 | 48.6 – 56.3 | MIS |
| Zambia | 2007 | 59.1 | 1830 | 1.8 | 55.5 – 62.7 | DHS |
| Zambia | 2008 | 58.1 | 1658 | 1.7 | 54.7 – 61.5 | MIS |
| Zambia | 2010 | 65.2 | 1595 | 2.4 | 60.5 – 69.9 | MIS |
| Zimbabwe | 2005 | 7.4 | 1642 | 1.0 | 5.5 – 9.4 | DHS |

Malaria in pregnancy interventions: IPTp or ITNs, whichever is higher

CAR: Central African Republic

DRC: Democratic republic of Congo

1. These estimates are pregnant women sleeping under an ITN the night before the survey; all others are IPTp received among women giving birth in the past 2 years

2. These standard errors are estimated using formula 1

3. These estimates were not specified as 2+ doses of SP received at ANC

4. Data available were only for Bioko Island
