# Supplementary material for: Estimates of child deaths prevented from malaria prevention scale-up in Africa 2001-2010
Source: Malar J. 2012 Mar 28;11:93. doi: 10.1186/1475-2875-11-93 (PMC3350413; doi:10.1186/1475-2875-11-93)

**Additional file 4: Figures of malaria prevention coverage estimates 2001-2010 for each country**

**Country specific estimates of malaria prevention in pregnancy (IPTp/ITNs) coverage in rural areas from 2000-2010.** The blue line is the predicted malaria in pregnancy prevention intervention scale-up. The red line is the scenario of the lowest coverage increase used for lower bound estimate for uncertainty; the green line is the scenario of the highest coverage increase used for the upper bound estimate for uncertainty. Circles represent MICS surveys, diamonds represent DHS surveys, triangles represent MIS surveys, and squares represent other types of household surveys.

Angola Benin


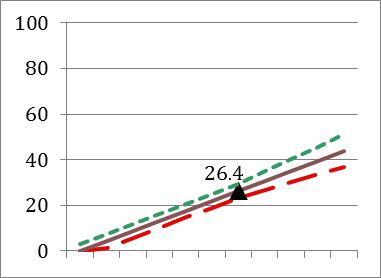

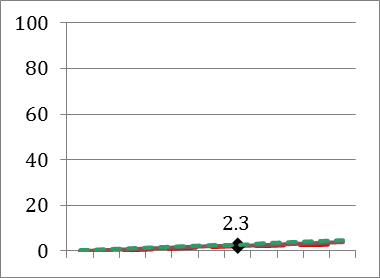


Burkina Faso Cameroon


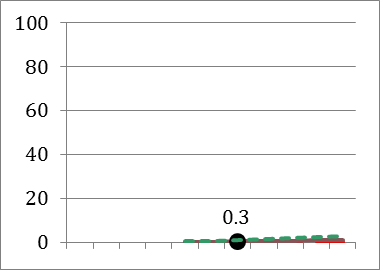

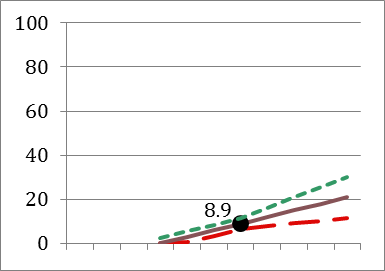


Central African Republic Chad


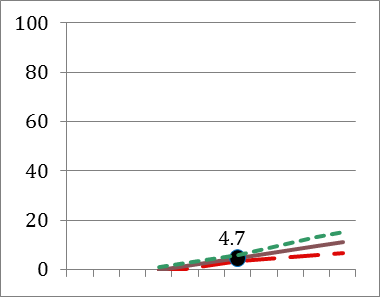

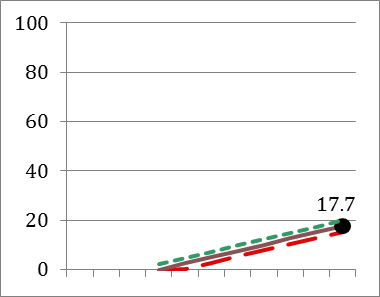


Congo (Brazzaville) Cote d’Ivoire


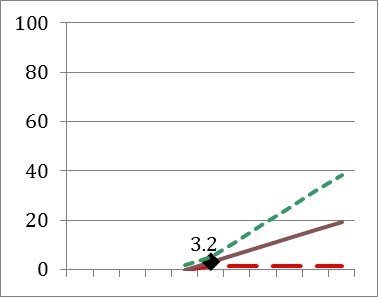

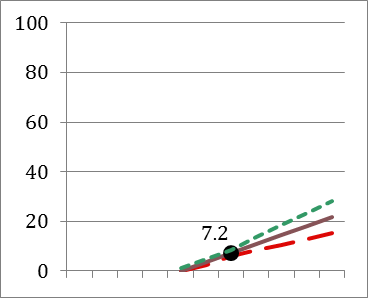


Democratic Republic of the Congo Equatorial Guinea


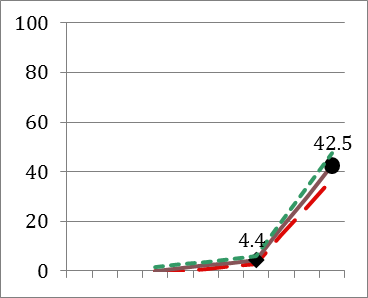

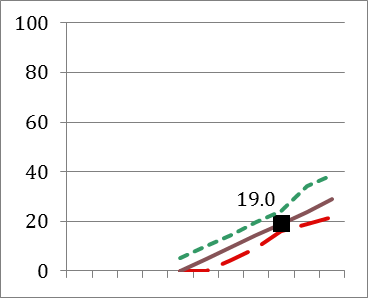


Gambia Ghana


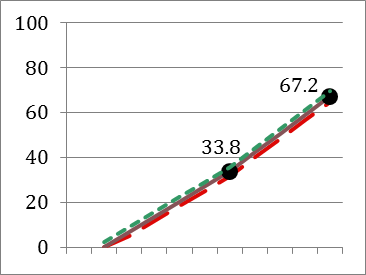

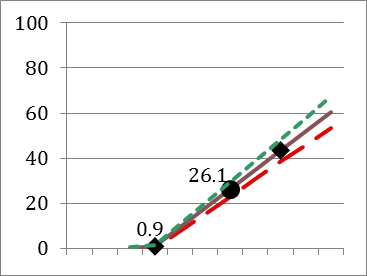


Guinea Guinea-Bissau


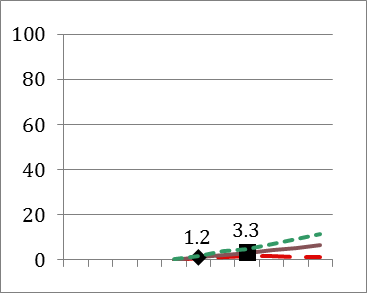

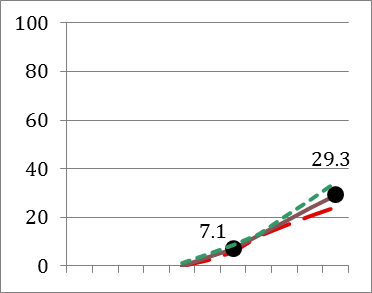


Kenya Liberia


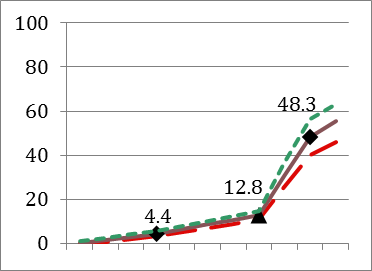

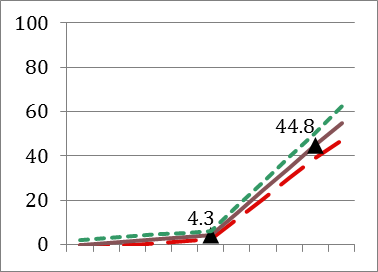


Madagascar Malawi


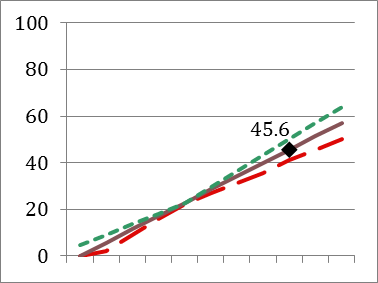

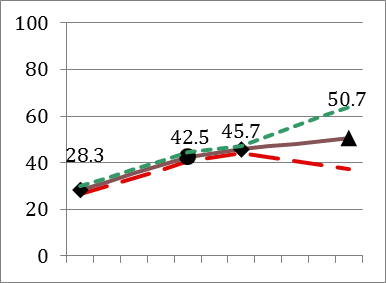


Mali Mozambique


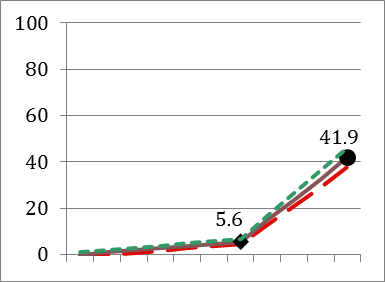

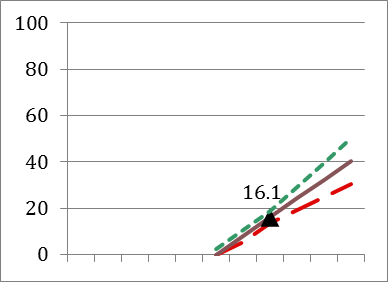


Niger Nigeria


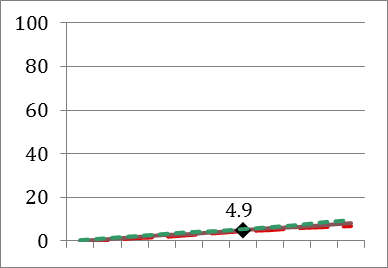

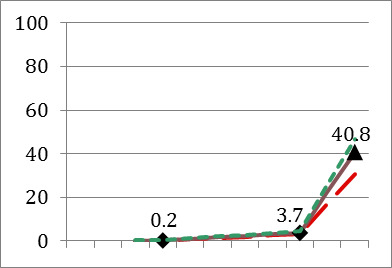


Rwanda Senegal


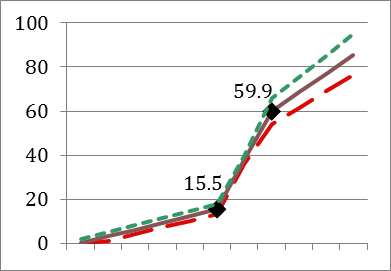

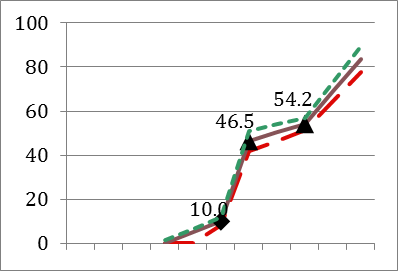


Sierra Leone Somalia


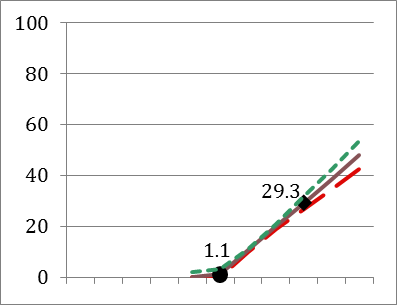

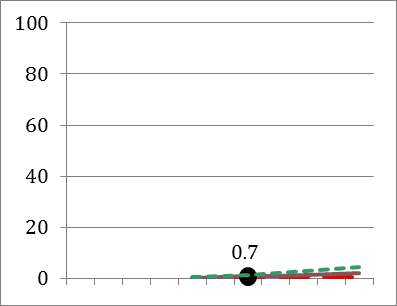


Sudan Tanzania


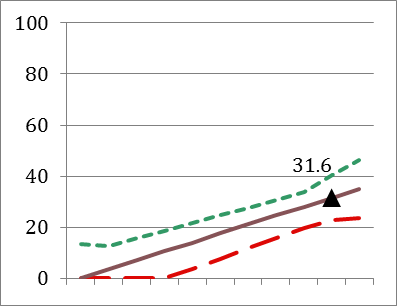

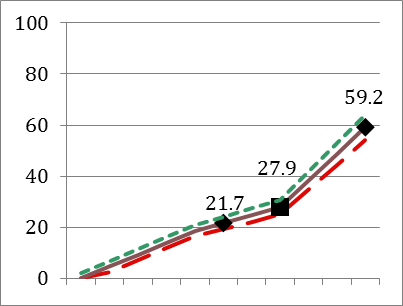


Togo Uganda


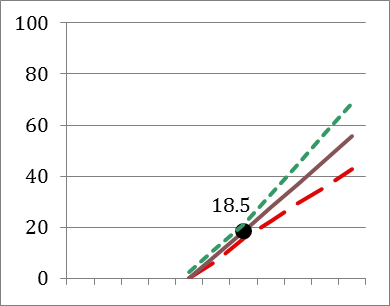

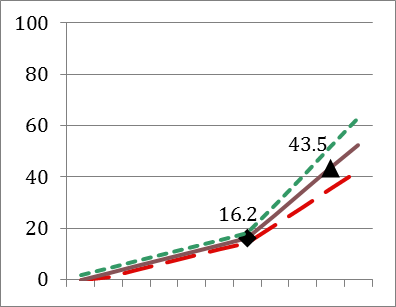


Zambia Zimbabwe


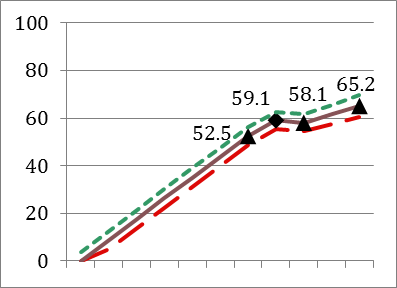

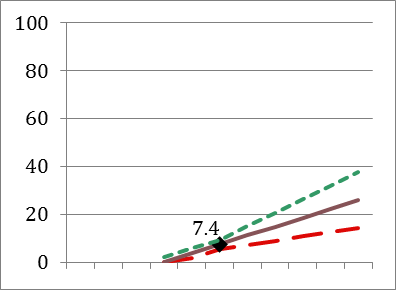

Supplement: Additional file 4 — Table of malaria in pregnancy intervention coverage estimates from household surveys 2000-2010. Table of malaria in pregnancy intervention coverage estimates from household surveys 2000-2010. [file 1475-2875-11-93-S4.DOC]
