# Supplementary material for: Estimates of child deaths prevented from malaria prevention scale-up in Africa 2001-2010
Source: Malar J. 2012 Mar 28;11:93. doi: 10.1186/1475-2875-11-93 (PMC3350413; doi:10.1186/1475-2875-11-93)
Supplement: Additional file 5 — Tables and additional details of the cost-effectiveness methods and results of sensitivity analysis. Tables and additional details of the cost-effectiveness methods and results of sensitivity analysis. [file 1475-2875-11-93-S5.DOC]

**Additional file 5: Tables and additional details of the cost-effectiveness methods and results of sensitivity analysis**

**Table 1: Results of a literature review on the cost of delivering ITNs and LLINs: studies in sub-Saharan Africa since 2005 (all figures in US$)**

|  | | | | **Economic Cost** | | | | |
| --- | --- | --- | --- | --- | --- | --- | --- | --- |
| **Channel of delivery** | **Country** | **Year** | **Source** | **Cost of delivery*** | **Commodity Cost**  **(3 year life)*** | **Total (Delivery + Commodity)*** | **Cost per**  **/ year of availability (year of protection)**** | **Cost of delivery**** |
| Antenatal clinics  (with charges for nets) | Burkina Faso | 2006 |  | 3.68 | 5.18 | 8.86 | 3.33 | 3.91 |
|  | Kenya (WHO) | 2007 |  | 2.52 | 6.17 | 8.69 | 3.17 | 2.60 |
|  | Malawi | 2005 |  | 1.01 | 2.19 | 3.20 | 1.24 | 1.11 |
|  | Tanzania | 2005 |  | 2.06 | 3.40 | 5.46 | 2.12 | 2.26 |
|  | Malawi | 2005 |  | 0.70 | 1.54 | 2.24 | 0.87 | 0.77 |
| Antenatal clinics  (nets provided free) | DRC | 2006 |  | 1.51 | 8.00 | 9.51 | 3.57 | 1.61 |
|  | Kenya | 2008 |  | 1.97 | 5.20 | 7.17 | 2.57 | 1.99 |
|  | Uganda | 2007 |  | 2.27 | 5.26 | 7.53 | 2.75 | 2.35 |
|  | Zanzibar | 2005 |  | 2.07 | 6.52 | 8.59 | 3.33 | 2.27 |
| Mass campaign  (nets provided free) | Uganda | 2007 |  | 1.23 | 5.74 | 6.97 | 2.55 | 1.27 |
|  | Uganda | 2007 |  | 0.76 | 5.26 | 6.02 | 2.20 | 0.79 |
|  | Uganda | 2005–06 |  | 1.05 | 5.80 | 6.85 | 2.66 | 1.15 |
|  | Uganda | 2008–09 |  | 1.24 | 5.63 | 6.87 | 2.46 | 1.25 |
|  | Zanzibar | 2005–06 |  | 1.59 | 6.52 | 8.11 | 3.15 | 1.75 |
|  | Zanzibar | 2008–09 |  | 1.62 | 6.13 | 7.75 | 2.77 | 1.64 |
|  | Togo | 2004 |  | 1.25 | 4.32 | 5.57 | 2.23 | 1.42 |
|  | Eritrea | 2005 |  | 1.47 | 1.36 | 2.83 | 1.10 | 1.61 |
|  | Ghana | 2002 |  | 0.32 | 3.42 | 3.74 | 1.58 | 0.38 |
|  | Tanzania | 2009 |  | 1.83 | 5.24 | 7.07 | 2.50 | 1.83 |
| Retail sales | Burkina Faso | 2006 |  | 3.69 | 5.13 | 8.82 | 3.31 | 3.92 |
|  | Kenya | 2008 |  | 2.06 | 5.20 | 7.26 | 2.60 | 2.08 |
|  | Tanzania | 2006 |  | 4.66 | 2.98 | 7.64 | 2.87 | 4.95 |
|  | Senegal | 2005 |  | 3.64 |  |  |  | 3.99 |
| **Median Value** | |  |  |  |  |  | **2.57** | **1.64** |

*Nominal US$; **2009 US$

**Table 2**: Results of one-way sensitivity analysis

| **Variable** | **Baseline Value** | **Change** | **Result After change cost per DALY** | **Interpretation (Cost-effective according to CMH guidelines)** | **Justification** |
| --- | --- | --- | --- | --- | --- |
| Discount Rate | 3% | Raised to 10% | Increased from USD 111 to USD 118 | Cost-effective | Represents a high discount rate for standard health economic analysis |
| Number of discounted lives Saved by ITNs (2006-2009) | 475,826 | Reduced to 326,233 | Increased from USD 111 to USD 162 | Cost-effective | Lowest estimate for period 2006-2009 produced by LiST Model |
| Cost of ITN delivery | USD 1.64 | Raised to USD 3.96 | Increased from USD 111 to USD 155 | Cost-effective | Median estimate for retail sales delivery cost (highest subgroup in lit review) |
| Cost of LLIN | USD 5.44 | Raised to USD 7.89 | Increased from USD 111 to USD 147 | Cost-effective | Highest price paid per lot of LLIN in GFATM PRQ database |
| Net lifetime | 3 years | Reduced to one year | Increased from USD 111 to USD 114 | Cost-effective | Lowest time period over which costs can be annualized |
| Net lifetime | 3 years | Increased to five years | Reduced from USD 111 to USD 100 | Cost-effective | Upper bound of planning for net replacement |
